# Supplementary material for: Self-Assembled Peptide Habitats to Model Tumor Metastasis
Source: Gels. 2022 May 25;8(6):332. doi: 10.3390/gels8060332 (PMC9223161; doi:10.3390/gels8060332)
Supplement: Supplementary file 1 [file gels-08-00332-s001.zip › gels-1721285-supplementary.pdf]

gels MDPI

Supplementary Materials

Self-Assembled Peptide Habitats to Model Tumor Metastasis

Noora Al Balushi [1], Mitchell Boyd-Moss [2,3,4], Rasika M. Samarasinghe [3,5], Aaqil Rifai [2,3,5], Stephanie J. Franks [6], Kate Firipis [2,4], Benjamin M. Long [7], Ian A. Darby [1], David R. Nisbet [6,8,9,10], Dodie Pouniotis [1,*] and Richard J. Williams [2,3,4,5,*]

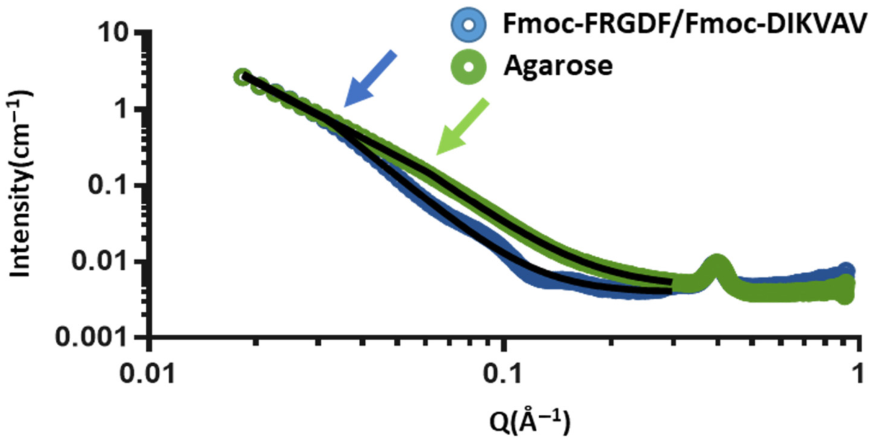

Figure S1. Fitting of scattering curves using a two-power model to determine mesh size (arrows indicate the power intercept).

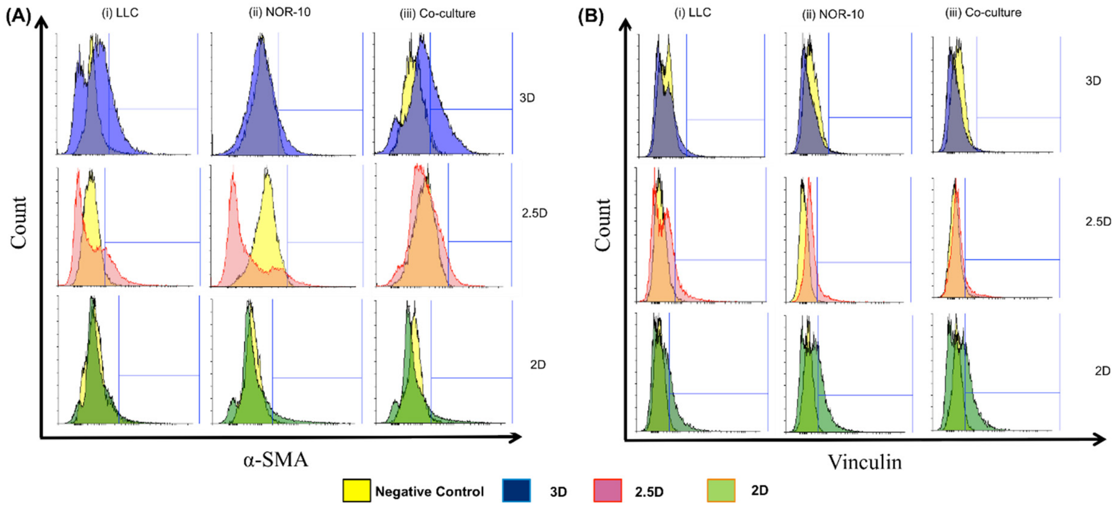

Figure S2. Flow cytometry of (A) α-SMA and (B) vinculin expression measured in (i) LLC, (ii) NOR-10 and (iii) LLC+NOR-10 co-culture after 72 hours encapsulation within Fmoc-SAP hydrogel (3D) compared to 2.5D spheroids in media and 2D cell culture.

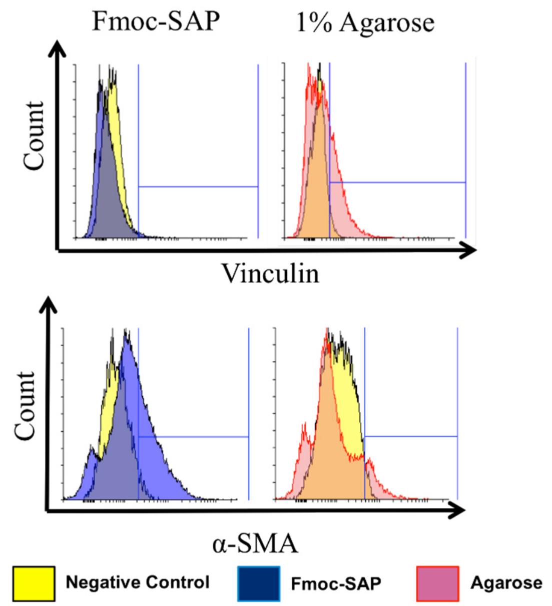

Figure S3. Flow cytometry analysis of vinculin and α-SMA expression in co-culture spheroids within Fmoc-SAP hydrogels and 1% agarose.
